# Crystal structure of 2-(4-chloro­phen­yl)-3-(4-meth­oxy­phen­yl)-3-(methyl­sulfanyl)­acrylo­nitrile

**DOI:** 10.1107/S1600536814021576

**Published:** 2014-10-11

**Authors:** Shamantha Kumar, Amar A. Hosamani, A. C. Vinayaka, M. P. Sadashiva, B. H. Doreswamy

**Affiliations:** a Department of Physics, SJB Institute of Technology, Kengeri, Bangalore 560 060, India; b Solid State and Structural Chemistry Unit, Indian Institute of Science, Bangalore 560 012, India; c Department of Studies in Chemistry, Manasagangotri, University of Mysore, Mysore 570 006, India

**Keywords:** crystal structure, acrylo­nitrile, C—H⋯π inter­actions, biological activity, pharmacological activity

## Abstract

In the title compound, C_17_H_14_ClNOS, the aromatic rings are inclined to one another by 64.22 (9)°. The acrylo­nitrile group (C=C—C N) is planar to within 0.003 (2) Å, with the S atom and the methyl C atom displaced from this plane by 0.2317 (6) and −0.637 (2) Å, respectively. In the crystal, mol­ecules are linked *via* pairs of C—H⋯π inter­actions, forming inversion dimers. There are no other significant inter­molecular inter­actions present.

## Related literature   

For the biological and pharmacological activities of acrylo­nitrile derivatives, see: Boëdec *et al.* (2008 ▶); Napolitano *et al.* (2001 ▶); Saczewski *et al.* (2004 ▶); Sommen *et al.* (2003 ▶). For related literature, see: Saufi & Ismail (2002 ▶); Urska *et al.* (2003 ▶).
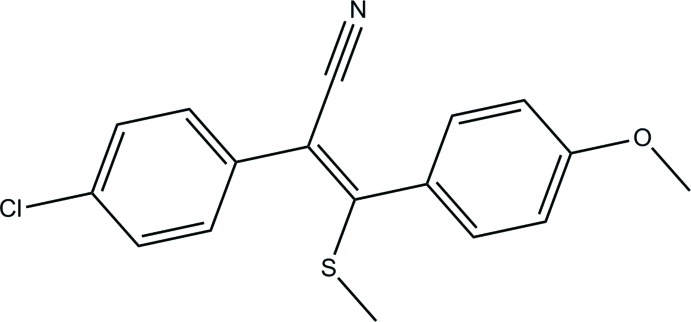


## Experimental   

### Crystal data  

C_17_H_14_ClNOS
*M* *_r_* = 315.81
Monoclinic, 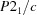

*a* = 8.3060 (4) Å
*b* = 10.5048 (6) Å
*c* = 17.9795 (9) Å
β = 100.598 (5)°
*V* = 1542.00 (14) Å^3^
*Z* = 4
Mo *K*α radiation
μ = 0.38 mm^−1^
*T* = 293 K
0.30 × 0.25 × 0.20 mm

### Data collection  

Bruker APEXII CCD area-detector diffractometer
6889 measured reflections
3537 independent reflections
2807 reflections with *I* > 2σ(*I*)
*R*_int_ = 0.028

### Refinement  

*R*[*F* ^2^ > 2σ(*F* ^2^)] = 0.046
*wR*(*F* ^2^) = 0.114
*S* = 1.04
3537 reflections
192 parameters
H-atom parameters constrained
Δρ_max_ = 0.26 e Å^−3^
Δρ_min_ = −0.26 e Å^−3^

### 

Data collection: *APEX2* (Bruker, 2009 ▶); cell refinement: *SAINT* (Bruker, 2009 ▶); data reduction: *SAINT*; program(s) used to solve structure: *SHELXS97* (Sheldrick, 2008 ▶); program(s) used to refine structure: *SHELXL97* (Sheldrick, 2008 ▶); molecular graphics: *PLATON* (Spek, 2009 ▶); software used to prepare material for publication: *SHELXL97*.

## Supplementary Material

Crystal structure: contains datablock(s) global, I. DOI: 10.1107/S1600536814021576/su2787sup1.cif

Structure factors: contains datablock(s) I. DOI: 10.1107/S1600536814021576/su2787Isup2.hkl

Supporting information file. DOI: 10.1107/S1600536814021576/su2787Isup3.cml

**Fig. 1.**
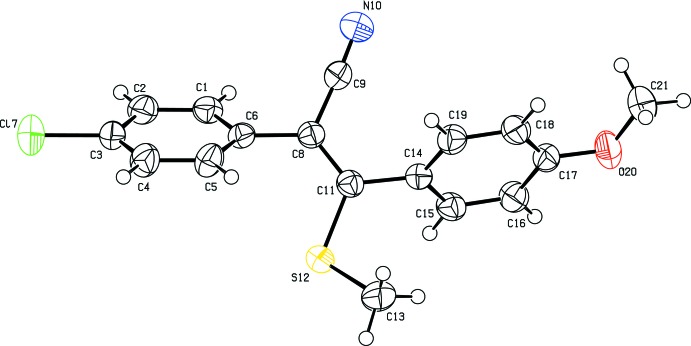
A view of the molecular structure of the title molecule, with atom labelling. Displacement ellipsoids are drawn at the 50% probability level.

**Fig. 2.**
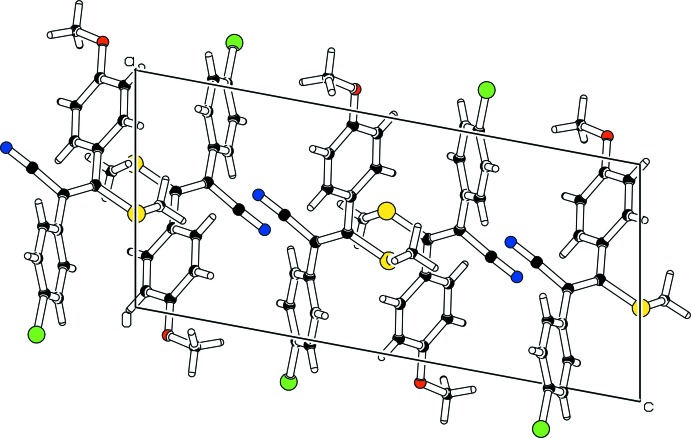
A view along the b axis of the crystal packing of the title compound.

. DOI: 10.1107/S1600536814021576/su2787fig1.tif
A view of the mol­ecular structure of the title mol­ecule, with atom labelling. Displacement ellipsoids are drawn at the 50% probability level.

b . DOI: 10.1107/S1600536814021576/su2787fig2.tif
A view along the *b* axis of the crystal packing of the title compound.

CCDC reference: 1026843

Additional supporting information:  crystallographic information; 3D view; checkCIF report

## Figures and Tables

**Table 1:** Hydrogen-bond geometry (, ) *Cg*1 is the centroid of the C1C6 ring.

*D*H*A*	*D*H	H*A*	*D* *A*	*D*H*A*
C15H15*Cg*1^i^	0.93	2.96	3.739(2)	142

Symmetry code: (i) 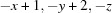.

## supplementary crystallographic information

### S1. Experimental

To a stirred suspension of NaH (0.45 g, 11.0 mmol, 60% suspension in oil) in dry THF (25 ml), a solution of (4-chloro-phenyl)-acetonitrile (0.75 g, 5.0 mmol) and 4-methoxy-dithiobenzoic acid methyl ester (0.99 g, 5.0 mmol) in dry THF (50 ml) was added drop wise at 273 K under a nitrogen atmosphere. The reaction mixture was stirred at room temperature for 4 h. It was again cooled to 273 K and methyl iodide (1.42 g, 10 mmol) was added drop wise. The reaction mixture was further stirred at room temperature for 4 h and poured into ice cold water (25 ml). The aqueous layer was extracted with CH_2_Cl_2_ (3 × 10 ml). The combined organic extracts were washed with water (1 × 20 ml), brine (1 × 20 ml), and dried over anhydrous Na_2_SO_4_. The solvent was evaporated under reduced pressure to give a crude product which was purified by silica gel column using EtOAc:hexane as eluent. Colourless block-like crystals were grown by dissolving the product in absolute ethanol followed by slow evaporation at room temperature.

### S2. Refinement

The C-bound H atoms were positioned geometrically and allowed to ride on their parent atoms: C–H = 0.93 - 0.96 Å, with *U*_iso_(H) = 1.5*U*_eq_(C) for methyl H atoms and = 1.2U_eq_(C) for other H atoms.

### Crystal data

**Table:**

C_17_H_14_ClNOS	*F*(000) = 656
*M**_r_* = 315.81	*D*_x_ = 1.360 Mg m^−^^3^
Monoclinic, *P*2_1_/*c*	Mo *K*α radiation, λ = 0.71073 Å
Hall symbol: -P 2ybc	Cell parameters from 3537 reflections
*a* = 8.3060 (4) Å	θ = 2.5–27.5°
*b* = 10.5048 (6) Å	µ = 0.38 mm^−^^1^
*c* = 17.9795 (9) Å	*T* = 293 K
β = 100.598 (5)°	Block, colourless
*V* = 1542.00 (14) Å^3^	0.30 × 0.25 × 0.20 mm
*Z* = 4	

### Data collection

**Table:**

Bruker APEXII CCD area-detector diffractometer	*R*_int_ = 0.028
ω and φ scans	θ_max_ = 27.5°, θ_min_ = 2.5°
6889 measured reflections	*h* = −10→6
3537 independent reflections	*k* = −13→5
2807 reflections with *I* > 2σ(*I*)	*l* = −23→23

### Refinement

**Table:**

Refinement on *F*^2^	Primary atom site location: structure-invariant direct methods
Least-squares matrix: full	Secondary atom site location: difference Fourier map
*R*[*F*^2^ > 2σ(*F*^2^)] = 0.046	Hydrogen site location: inferred from neighbouring sites
*wR*(*F*^2^) = 0.114	H-atom parameters constrained
*S* = 1.04	*w* = 1/[σ^2^(*F*_o_^2^) + (0.045*P*)^2^ + 0.3209*P*] where *P* = (*F*_o_^2^ + 2*F*_c_^2^)/3
3537 reflections	(Δ/σ)_max_ < 0.001
192 parameters	Δρ_max_ = 0.26 e Å^−^^3^
0 restraints	Δρ_min_ = −0.26 e Å^−^^3^

### Special details

**Table:**

Geometry. Bond distances, angles *etc*. have been calculated using the rounded fractional coordinates. All su's are estimated from the variances of the (full) variance-covariance matrix. The cell e.s.d.'s are taken into account in the estimation of distances, angles and torsion angles
Refinement. Refinement on *F*^2^ for ALL reflections except those flagged by the user for potential systematic errors. Weighted *R*-factors *wR* and all goodnesses of fit *S* are based on *F*^2^, conventional *R*-factors *R* are based on *F*, with *F* set to zero for negative *F*^2^. The observed criterion of *F*^2^ > σ(*F*^2^) is used only for calculating -*R*-factor-obs *etc*. and is not relevant to the choice of reflections for refinement. *R*-factors based on *F*^2^ are statistically about twice as large as those based on *F*, and *R*-factors based on ALL data will be even larger.

### Fractional atomic coordinates and isotropic or equivalent isotropic displacement parameters (Å^2^)

**Table:**

	*x*	*y*	*z*	*U*_iso_*/*U*_eq_	
Cl7	−0.19319 (7)	1.21573 (7)	−0.19619 (4)	0.0708 (2)	
S12	0.39592 (6)	0.84311 (6)	0.00222 (3)	0.0463 (2)	
O20	1.09261 (17)	0.59095 (16)	−0.06455 (9)	0.0603 (6)	
N10	0.5710 (2)	0.9039 (2)	−0.25595 (10)	0.0575 (7)	
C1	0.2687 (2)	1.09961 (19)	−0.18246 (10)	0.0396 (6)	
C2	0.1284 (2)	1.1742 (2)	−0.19521 (11)	0.0463 (6)	
C3	−0.0153 (2)	1.1236 (2)	−0.18024 (11)	0.0441 (6)	
C4	−0.0223 (2)	1.0011 (2)	−0.15428 (12)	0.0496 (7)	
C5	0.1178 (2)	0.9272 (2)	−0.14196 (11)	0.0452 (6)	
C6	0.2658 (2)	0.97624 (18)	−0.15536 (9)	0.0347 (5)	
C8	0.4179 (2)	0.89756 (18)	−0.14288 (10)	0.0346 (5)	
C9	0.5065 (2)	0.8996 (2)	−0.20479 (10)	0.0401 (6)	
C11	0.4820 (2)	0.83108 (18)	−0.07963 (10)	0.0353 (5)	
C13	0.4464 (3)	0.6919 (2)	0.04764 (12)	0.0570 (8)	
C14	0.6374 (2)	0.75884 (19)	−0.07423 (10)	0.0350 (5)	
C15	0.7767 (2)	0.7996 (2)	−0.02329 (10)	0.0402 (6)	
C16	0.9244 (2)	0.7402 (2)	−0.02164 (11)	0.0443 (6)	
C17	0.9379 (2)	0.6388 (2)	−0.06941 (11)	0.0406 (6)	
C18	0.8005 (2)	0.5942 (2)	−0.11771 (11)	0.0441 (6)	
C19	0.6513 (2)	0.6553 (2)	−0.11980 (11)	0.0429 (6)	
C21	1.1194 (3)	0.4982 (3)	−0.11813 (15)	0.0742 (10)	
H1	0.36640	1.13300	−0.19230	0.0470*	
H2	0.13140	1.25680	−0.21350	0.0550*	
H4	−0.12060	0.96800	−0.14510	0.0600*	
H5	0.11330	0.84410	−0.12460	0.0540*	
H13A	0.40050	0.62460	0.01420	0.0860*	
H13B	0.40220	0.68780	0.09330	0.0860*	
H13C	0.56320	0.68250	0.05960	0.0860*	
H15	0.76880	0.86710	0.00940	0.0480*	
H16	1.01650	0.76820	0.01190	0.0530*	
H18	0.80790	0.52400	−0.14840	0.0530*	
H19	0.55900	0.62580	−0.15260	0.0520*	
H21A	1.06080	0.42180	−0.11070	0.1110*	
H21B	1.23430	0.47980	−0.11170	0.1110*	
H21C	1.08110	0.53000	−0.16830	0.1110*	

### Atomic displacement parameters (Å^2^)

**Table:**

	*U*^11^	*U*^22^	*U*^33^	*U*^12^	*U*^13^	*U*^23^
Cl7	0.0571 (3)	0.0760 (5)	0.0789 (4)	0.0308 (3)	0.0114 (3)	0.0093 (4)
S12	0.0507 (3)	0.0523 (3)	0.0385 (3)	0.0136 (2)	0.0150 (2)	0.0035 (2)
O20	0.0451 (8)	0.0666 (11)	0.0699 (10)	0.0192 (7)	0.0125 (7)	−0.0035 (9)
N10	0.0579 (11)	0.0707 (14)	0.0479 (10)	0.0082 (10)	0.0199 (8)	0.0068 (10)
C1	0.0436 (10)	0.0401 (11)	0.0362 (9)	−0.0011 (8)	0.0104 (8)	0.0014 (9)
C2	0.0594 (12)	0.0383 (11)	0.0421 (10)	0.0073 (9)	0.0120 (9)	0.0045 (9)
C3	0.0447 (10)	0.0491 (12)	0.0376 (10)	0.0149 (9)	0.0049 (8)	−0.0006 (9)
C4	0.0348 (9)	0.0585 (14)	0.0548 (12)	0.0010 (9)	0.0065 (8)	0.0081 (11)
C5	0.0393 (10)	0.0412 (12)	0.0532 (11)	−0.0006 (8)	0.0033 (8)	0.0103 (10)
C6	0.0369 (9)	0.0369 (10)	0.0293 (8)	0.0019 (8)	0.0033 (7)	−0.0004 (8)
C8	0.0344 (9)	0.0353 (10)	0.0336 (9)	−0.0007 (7)	0.0051 (7)	−0.0027 (8)
C9	0.0374 (9)	0.0437 (12)	0.0377 (10)	0.0035 (8)	0.0033 (8)	0.0013 (9)
C11	0.0360 (9)	0.0346 (10)	0.0351 (9)	−0.0002 (7)	0.0064 (7)	−0.0039 (8)
C13	0.0608 (13)	0.0651 (16)	0.0479 (12)	0.0059 (11)	0.0171 (10)	0.0161 (11)
C14	0.0368 (9)	0.0365 (10)	0.0321 (8)	0.0022 (7)	0.0078 (7)	0.0015 (8)
C15	0.0423 (10)	0.0408 (11)	0.0370 (9)	−0.0008 (8)	0.0063 (8)	−0.0078 (9)
C16	0.0358 (9)	0.0521 (13)	0.0437 (10)	−0.0014 (9)	0.0036 (8)	−0.0044 (10)
C17	0.0405 (10)	0.0420 (12)	0.0409 (10)	0.0071 (8)	0.0118 (8)	0.0077 (9)
C18	0.0552 (11)	0.0366 (11)	0.0405 (10)	0.0078 (9)	0.0091 (9)	−0.0052 (9)
C19	0.0434 (10)	0.0434 (12)	0.0396 (10)	0.0022 (9)	0.0013 (8)	−0.0049 (9)
C21	0.0776 (17)	0.0797 (19)	0.0691 (15)	0.0403 (15)	0.0239 (13)	0.0036 (15)

### Geometric parameters (Å, º)

**Table:**

Cl7—C3	1.745 (2)	C15—C16	1.372 (3)
S12—C11	1.7550 (18)	C16—C17	1.386 (3)
S12—C13	1.800 (2)	C17—C18	1.383 (3)
O20—C17	1.368 (2)	C18—C19	1.390 (3)
O20—C21	1.416 (3)	C1—H1	0.9300
N10—C9	1.147 (2)	C2—H2	0.9300
C1—C2	1.388 (3)	C4—H4	0.9300
C1—C6	1.386 (3)	C5—H5	0.9300
C2—C3	1.378 (3)	C13—H13A	0.9600
C3—C4	1.374 (3)	C13—H13B	0.9600
C4—C5	1.382 (3)	C13—H13C	0.9600
C5—C6	1.394 (2)	C15—H15	0.9300
C6—C8	1.492 (2)	C16—H16	0.9300
C8—C9	1.443 (2)	C18—H18	0.9300
C8—C11	1.357 (3)	C19—H19	0.9300
C11—C14	1.485 (2)	C21—H21A	0.9600
C14—C15	1.404 (2)	C21—H21B	0.9600
C14—C19	1.379 (3)	C21—H21C	0.9600
			
C11—S12—C13	102.71 (10)	C2—C1—H1	119.00
C17—O20—C21	118.21 (17)	C6—C1—H1	119.00
C2—C1—C6	121.16 (16)	C1—C2—H2	121.00
C1—C2—C3	118.77 (19)	C3—C2—H2	121.00
Cl7—C3—C2	119.45 (16)	C3—C4—H4	120.00
Cl7—C3—C4	119.20 (14)	C5—C4—H4	120.00
C2—C3—C4	121.34 (17)	C4—C5—H5	120.00
C3—C4—C5	119.57 (17)	C6—C5—H5	120.00
C4—C5—C6	120.52 (19)	S12—C13—H13A	109.00
C1—C6—C5	118.62 (17)	S12—C13—H13B	109.00
C1—C6—C8	120.12 (15)	S12—C13—H13C	110.00
C5—C6—C8	121.24 (17)	H13A—C13—H13B	109.00
C6—C8—C9	114.44 (15)	H13A—C13—H13C	109.00
C6—C8—C11	127.02 (16)	H13B—C13—H13C	109.00
C9—C8—C11	118.48 (16)	C14—C15—H15	120.00
N10—C9—C8	176.9 (2)	C16—C15—H15	120.00
S12—C11—C8	120.54 (14)	C15—C16—H16	120.00
S12—C11—C14	117.73 (13)	C17—C16—H16	120.00
C8—C11—C14	121.34 (16)	C17—C18—H18	120.00
C11—C14—C15	119.27 (17)	C19—C18—H18	120.00
C11—C14—C19	122.06 (16)	C14—C19—H19	119.00
C15—C14—C19	118.62 (16)	C18—C19—H19	119.00
C14—C15—C16	120.12 (18)	O20—C21—H21A	109.00
C15—C16—C17	120.68 (17)	O20—C21—H21B	110.00
O20—C17—C16	115.16 (16)	O20—C21—H21C	109.00
O20—C17—C18	124.96 (18)	H21A—C21—H21B	109.00
C16—C17—C18	119.88 (17)	H21A—C21—H21C	109.00
C17—C18—C19	119.30 (19)	H21B—C21—H21C	109.00
C14—C19—C18	121.31 (17)		
			
C13—S12—C11—C8	152.50 (16)	C9—C8—C11—S12	170.97 (14)
C13—S12—C11—C14	−34.67 (17)	C9—C8—C11—C14	−1.6 (3)
C21—O20—C17—C18	7.3 (3)	C6—C8—C11—S12	−5.9 (3)
C21—O20—C17—C16	−172.2 (2)	C6—C8—C11—C14	−178.51 (17)
C2—C1—C6—C8	179.37 (17)	S12—C11—C14—C15	−60.3 (2)
C2—C1—C6—C5	0.9 (3)	C8—C11—C14—C19	−65.1 (3)
C6—C1—C2—C3	0.2 (3)	S12—C11—C14—C19	122.17 (18)
C1—C2—C3—Cl7	−179.80 (15)	C8—C11—C14—C15	112.5 (2)
C1—C2—C3—C4	−1.0 (3)	C11—C14—C19—C18	175.46 (18)
C2—C3—C4—C5	0.8 (3)	C15—C14—C19—C18	−2.1 (3)
Cl7—C3—C4—C5	179.59 (16)	C11—C14—C15—C16	−174.94 (18)
C3—C4—C5—C6	0.3 (3)	C19—C14—C15—C16	2.7 (3)
C4—C5—C6—C8	−179.57 (18)	C14—C15—C16—C17	−0.7 (3)
C4—C5—C6—C1	−1.1 (3)	C15—C16—C17—C18	−2.0 (3)
C1—C6—C8—C9	−47.0 (2)	C15—C16—C17—O20	177.50 (18)
C5—C6—C8—C11	−51.5 (3)	O20—C17—C18—C19	−176.86 (19)
C1—C6—C8—C11	130.0 (2)	C16—C17—C18—C19	2.6 (3)
C5—C6—C8—C9	131.45 (18)	C17—C18—C19—C14	−0.5 (3)

### Hydrogen-bond geometry (Å, º)

Cg1 is the centroid of the C1–C6 ring.

**Table:**

*D*—H···*A*	*D*—H	H···*A*	*D*···*A*	*D*—H···*A*
C15—H15···*Cg*1^i^	0.93	2.96	3.739 (2)	142

Symmetry code: (i) −*x*+1, −*y*+2, −*z*.
