# Supplementary figures and images for: Crystal structure of 2-(4-chloro­phen­yl)-3-(4-meth­oxy­phen­yl)-3-(methyl­sulfanyl)­acrylo­nitrile
Source: Acta Crystallogr Sect E Struct Rep Online. 2014 Oct 11;70(Pt 11):o1155. doi: 10.1107/S1600536814021576 (PMC4257267; doi:10.1107/S1600536814021576)

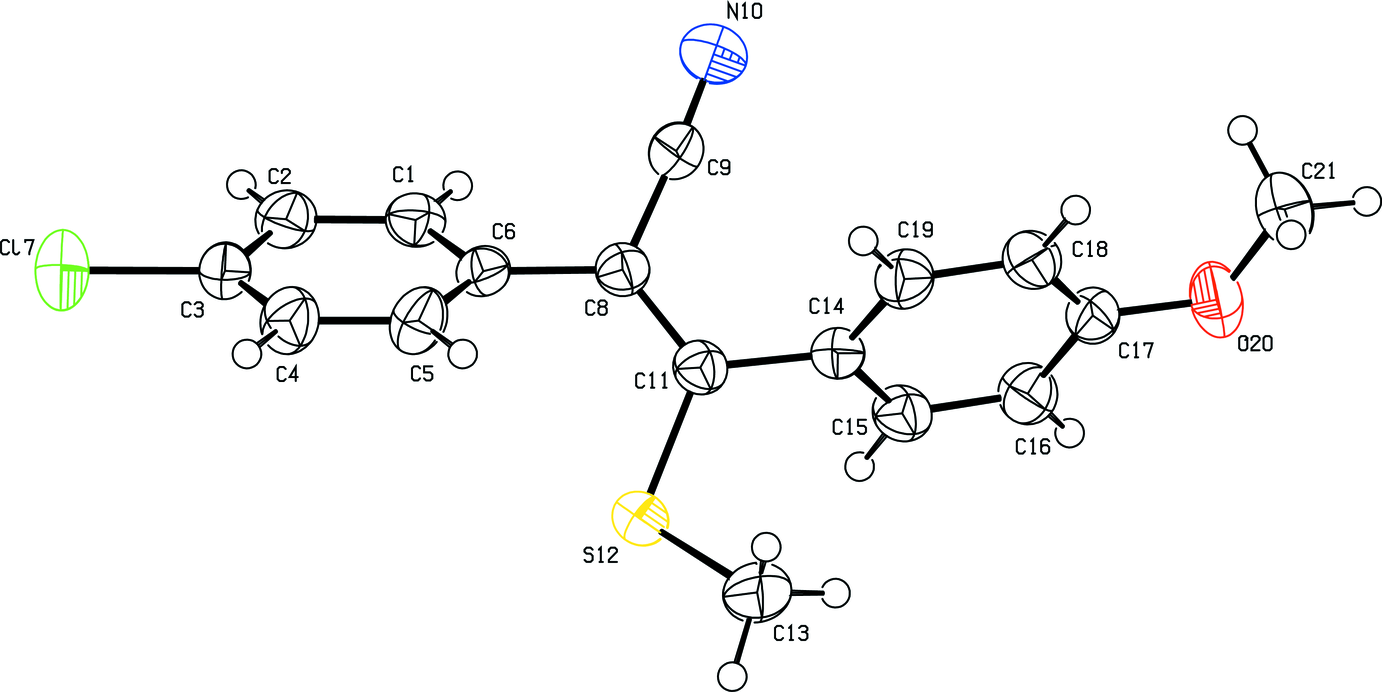

Supplement: Supplementary file 4 [file e-70-o1155-fig1.tif]

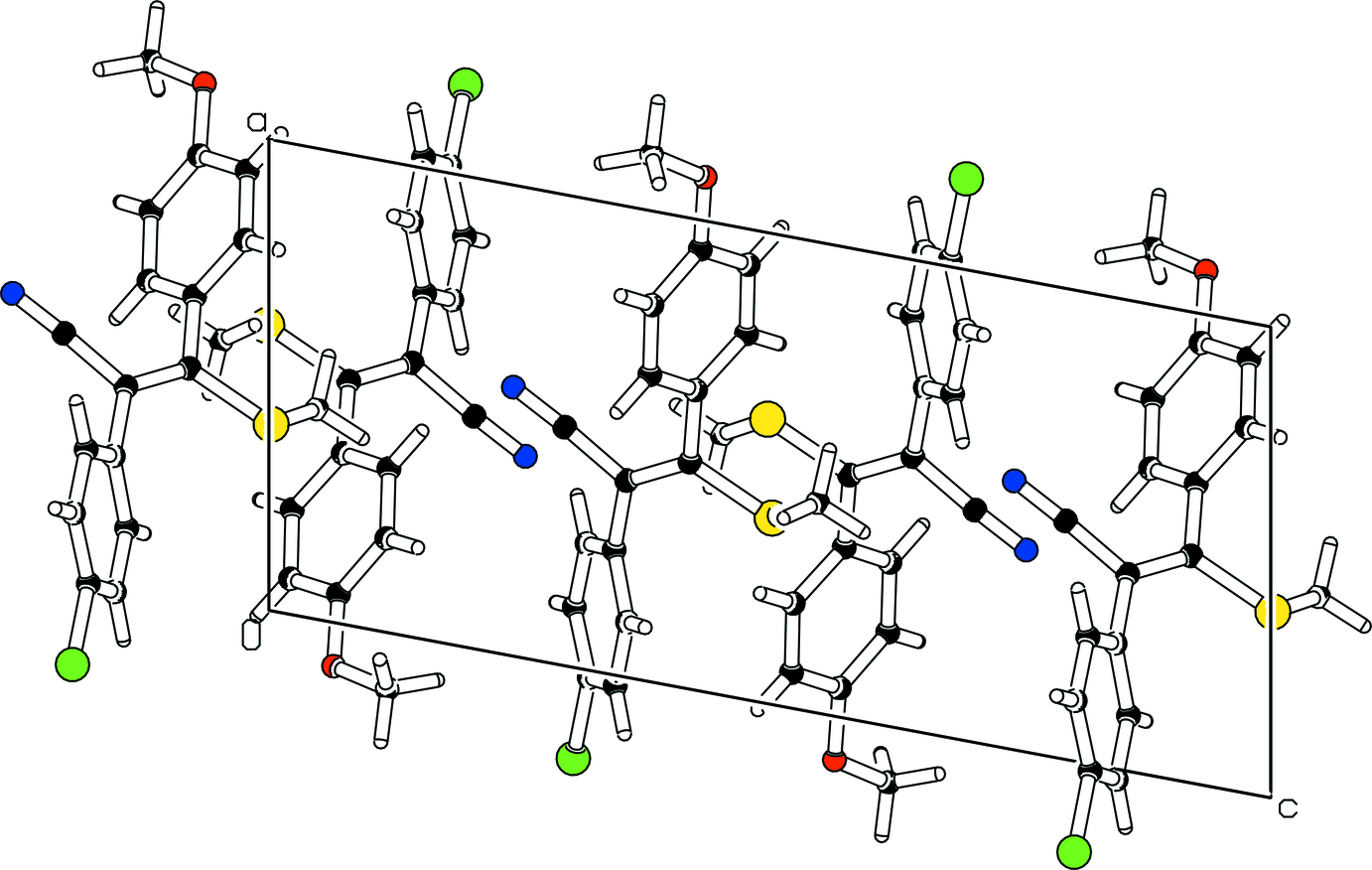

Supplement: Supplementary file 5 [file e-70-o1155-fig2.tif]
